# Supplementary material for: Learning curve and surgical outcome of robotic assisted colorectal surgery with ERAS program
Source: Sci Rep. 2022 Nov 29;12:20566. doi: 10.1038/s41598-022-24665-w (PMC9709162; doi:10.1038/s41598-022-24665-w)
Supplement: Supplementary file 1 — Supplementary Information 1. [file 41598_2022_24665_MOESM1_ESM.zip › LSC Raw data-Ñ[▒K/136/N_ñw╝╨░O▒Kñσ.pdf]

W52 / W52

臺 中 榮 民 總 醫 院

手術室護理記錄

[redacted]

基本資料

- 室別： 二醫手術室-31室，手術科別： CRS，手術類別： 常規，日期： 2021/09/01
- 進入時間： 0750 ； 麻醉開始時間： 0800 ； Time Out時間： 0905 ； 劃刀時間： 0905 ； 手術結束時間： 1315 送出時間： 1330 ；
- 麻醉方式： GE

  診斷： S colon ca.

  手術名稱： 1.Laparoscopic Anterior resection and anastomosis - malignant 2.Liver biopsy only3.4.

  手術局部用藥： NIL

  術前預防性抗生素：
  - 抗生素名稱一： Veterin 1GM IVD/由麻醉護理執行
  - 時間： 0900
  - 上次簽章: 彭馨慧

  ，追加預防性抗生素：
- 手術醫師： 林俊余
- 麻醉醫師： VS 施涵薰
-
  - 刷手護士A ： 林采儀，時間： 0750，接替刷手一： 彭馨慧，時間： 1130
  - 流動護士A ： 彭馨慧，時間： 0750，接替流動一： 朱詠暄，時間： 1100，接替流動二： 林采儀，時間： 1200

皮膚狀況

A-V Shunt： 無

術前： 不完整

部位(術前)：

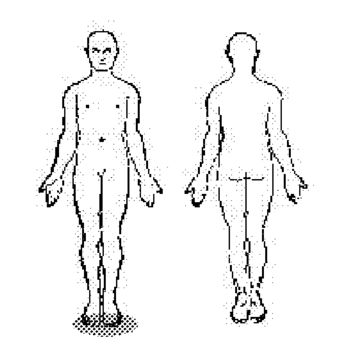

雙腳腳趾脫皮及擦傷，雙側大拇指紗布覆蓋

記錄者： 上次簽章: 彭馨慧

術後： 不完整

部位(術後)：

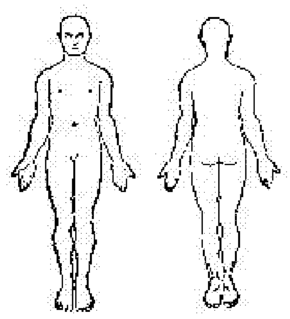

雙腳腳趾脫皮及擦傷，雙側大拇指紗布覆蓋

記錄者： 上次簽章: 林采儀

手術準備

手術臥式 單· ·手術臥式

-
    - 手術臥式： 截石臥式
    - 使用附件： 溫毯 38 ℃
    - 皮膚準備： 10%Aq Betadine , Hibiscrub , 2% Chlorhexidine
    - 電燒： 使用電燒: 電極片位置： 其它： 使用床上迴路電極墊 , 記錄者： 上次簽章: 彭馨慧

止血帶T： 無

植入物

- 植入物名稱/號碼/數量/部位： Ethicon 12mm ligaclip/20釘/pelvic
  Ethicon EndoGIA 60/ 2釘/colon
  hyallorbarrigel/壹/pelvic
  Ethicon CDH29A/壹/colon

, 記錄者： 上次簽章: 林采儀

標本送檢

- 細菌培養 無
- 細胞學 無
- 冰凍切片 無
- 病理標本
  組織名稱一：s-colon
  ,
  組織名稱二：liver S2
  ,
  組織名稱三：IMA root
  , 更多組織名稱
    - 組織名稱四：mesentery LN
    - 組織名稱五：distal cutend
- 截肢 無

標本交付 標本： 壹 袋 , 標本： 零 盒 , 標本： 零 瓶 , 細胞學： 零 管 , Culture Tube： 零 管 , 被交付醫師： R 林志安 ,

- 記錄者： 上次簽章: 林采儀
- 時間： 1250

傷口術後敷料

傷口敷料： 傷口敷料： 無

- 請於圖上標示部位：

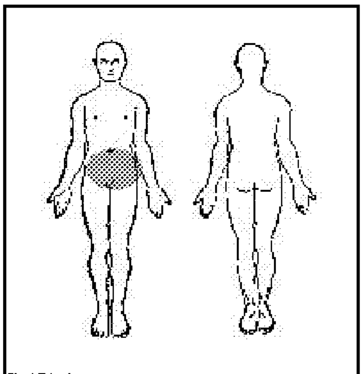

- 傷口敷料： menpor覆蓋

導管

導管	天數	導管資訊	插入日期	插入時間	更換日期	拔除日期	拔除時間	拔除原因	預計拔除/更換	預計拔除/更換日期
Foley(1)	1	號碼:16 Fr.; 部位:膀胱;	2021/09/01	0835						
Hemo Vac(1)	1	號碼:15 Fr.; 部位:骨盆腔;	2021/09/01	1150						
A-line (1)	1	號碼:20號 ; 部位:左手;	2021/09/01	0835						
Sump NG Tube(1)	1	號碼:16 Fr.; 部位:胃;	2021/09/01	0840						

用物計數

- 手術中出血量： minimal c.c.
- 手術中尿量： 700 c.c.
-
  - 其它： 無

用物計數： 單一切口

- 1.器械： 正確，2.縫針： 正確，3.刀片： 正確，4.紗布： 正確，5.腹部墊： 未使用，6.紗球： 未使用，7.PEANUTS ： 未使用，8.紗條： 未使用，9.絲帶： 未使用，10.針頭： 未使用，11.Vessel loop： 未使用，12.Boots： 未使用，13.Jelco： 未使用，14.棉條： 未使用，
  - 第一次：
    - 刷手護士 ： 彭馨慧
    - 流動護士： 上次簽章: 林采儀
    - 時間： 1225

  ,
  - 第二次：
    - 刷手護士 ： 彭馨慧
    - 流動護士： 上次簽章: 林采儀
    - 時間： 1235

  ,
  - 第三次：
    - 刷手護士 ： 彭馨慧
    - 流動護士： 上次簽章: 林采儀
    - 時間： 1250

備註

備註：NIL

- 記錄者： 林采儀
- 時間： 1330

填寫護理人員 林采儀

正式摘要

W52 / W52

doctorRank:B
